# Hesperidin ameliorates hypobaric hypoxia-induced retinal impairment through activation of Nrf2/HO-1 pathway and inhibition of apoptosis

**DOI:** 10.1038/s41598-020-76156-5

**Published:** 2020-11-10

**Authors:** Xiaorong Xin, Yanrong Li, Haiping Liu

**Affiliations:** Department of Ophthalmology, Sichuan Provincial People’s Hospital, University of Electronic Science and Technology of China, Chengdu, 610072 Sichuan Province China

**Keywords:** Medical research, Experimental models of disease

## Abstract

High-altitude retinopathy is initiated by hypobaric hypoxia and characterized by retinal functional changes, but the precise cellular and molecular mechanisms that mediate this dysfunction remain unclear. The aim of our investigation is to determine the protective efficacy of hesperidin (HSD) on the hypobaric hypoxia-induced damage to the retina. Experiment rats were randomly grouped as the control, hypobaric hypoxia group and HSD intervention group. The hypobaric hypoxia and the HSD intervention groups were maintained in a low-pressure oxygen cabin. We found that hypobaric hypoxia dramatically reduced nuclear factor erythroid 2-related factor 2 (Nrf2)/heme oxygenase-1(HO-1) levels, induced an elevation in immunostaining of TUNEL-positive cells. Hypobaric hypoxia exposure resulted in the increase of Bcl-2, decrease of caspase3 and caspase9 expression as well as Bax level. HSD protected the retina from hypobaric hypoxia-caused impairment by enhancing Nrf2 and HO-1 activation, attenuating apoptotic caspases levels, and reducing Bax and preserving Bcl-2 expression. Additionally, oxidative stress increased poly (ADP-ribose) polymerase 1 (PARP1) and suppressed ciliary neurotrophic factor (CNTF) level, HSD treatment reverted this effect by down-regulation of PARP1 and up-regulation of CNTF expression. Taken together, our findings implicate that HSD exerts a protective role in response to hypobaric hypoxia stress by activating Nrf2/HO-1 pathway and inhibiting apoptosis.

## Introduction

High attitude has a potential influence on human health due to the inadequacy of oxygen for the body’s metabolic requirements. Exposure to low barometric pressure initiates a series of physiologic changes such as increased cardiac output and ventilation, and is therefore susceptible to numerous acute altitude illnesses such as acute mountain sickness, high-altitude cerebral edema, and high-altitude pulmonary edema^1–3^. High-altitude retinopathy (HAR) is considered to be a retinal vascular decompensation associated with hypoxia. The dysfunction of retina such as optic disk swelling, vitreous hemorrhages, retinal capillary leakage, and retinal hemorrhages has been reported after high altitude-exposure^4–6^. However, the detail mechanism of HAR remains unknown. Our previous findings indicate that hypobaric hypoxia poses a pathological impact on the retina^7^. Visiting lowlanders are more likely to be susceptible to retinal dysfunction following exposure to high altitude and low barometric pressure.

Flavonoids, one of the most common polyphenols, are widely distributed in plants. Hesperidin (HSD) is a flavanone glycoside and has been found abundantly in citrus fruits. HSD exhibits beneficial medical effects in the regulation of biological and cellular process such as antioxidant, anti-inflammatory, anti-carcinogenic, antiallergic and neuropharmacological properties^8^. Especially, there is a noticeable body of evidence reveals that HSD mediates the neuroprotective effect through antioxidant, anti-inflammatory activities, calcium ion regulation, improvement of neuronal energy metabolism and reversing mitochondrial dysfunction^9–12^.

Emerging findings have been demonstrated the certain beneficial effects of HSD in various pathological condition, however less study has explored the role of HSD involved in hypobaric hypoxia-induced retinal impairment. In the current study, we investigated whether HSD supplementation can suppress oxidative stress-mediated apoptosis in the retina and if the activation of nuclear factor erythroid 2-related factor 2 (Nrf2)/heme oxygenase-1(HO-1) antioxidant pathway contributes to the protective effects of HSD to against hypobaric hypoxia-caused retinal injury .

## Results

### HSD treatment reversed hypobaric hypoxia-induced down-regulation of Nrf2 and HO-1 in the retina

**Figure 1:**
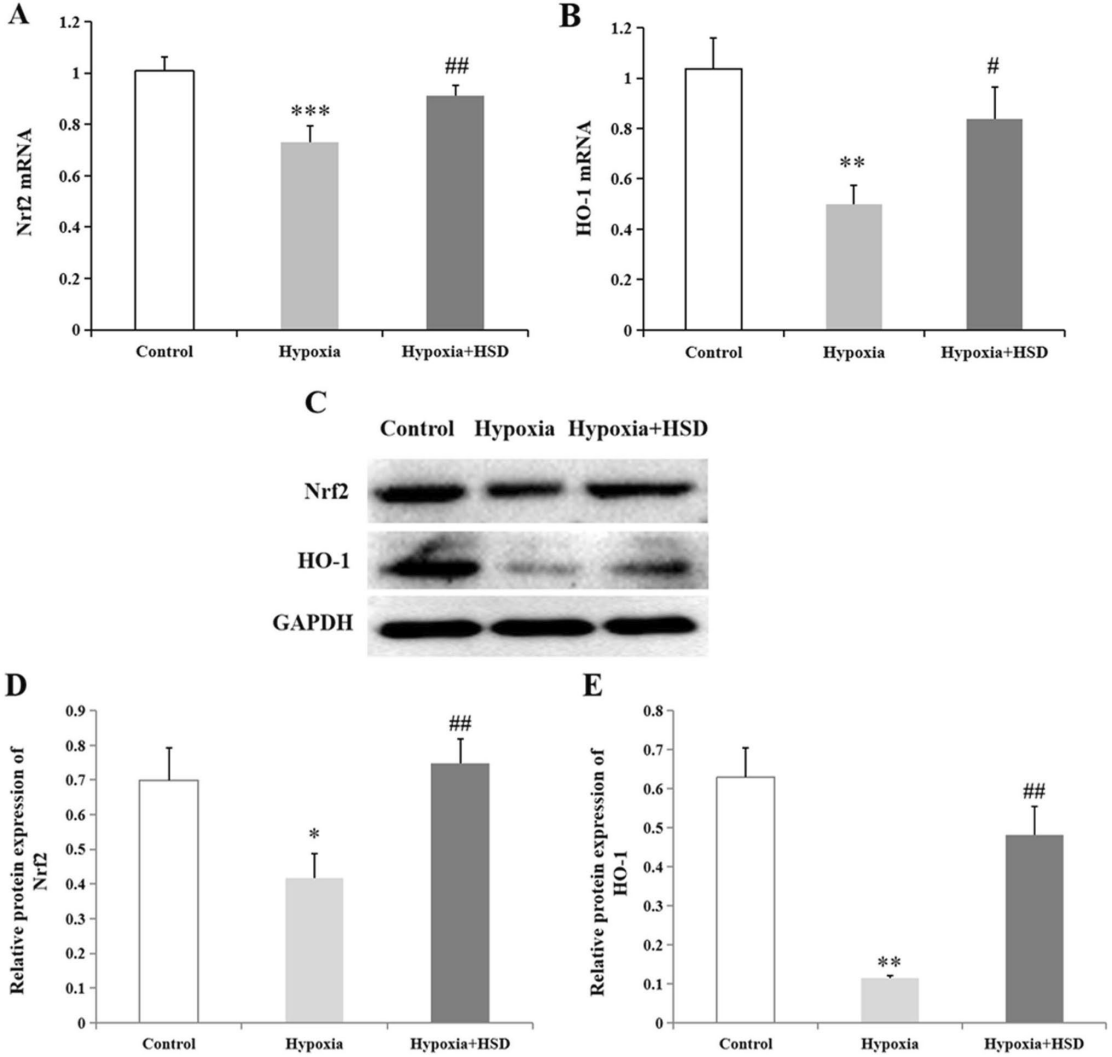
Real-time PCR and western blot analysis for Nrf2 and HO-1. Nrf2 mRNA (**A**) and HO-1 mRNA (**B**) expression in rat retina of control, hypobaric hypoxia and hesperidin intervention (Hypoxia + HSD) groups. The protein levels of Nrf2 and HO-1 were evaluated and GAPDH was used to ensure equal loading (**C**). (**D**,**E**) Densitometric analysis of proteins (D:Nrf2; E:HO-1). Results are presented as mean ± SEM (n = 6). *p < 0.05, **p < 0.01, ***p < 0.001, versus control; ^#^*p* < 0.05, ^##^*p* < 0.01, versus hypobaric hypoxia group. The uncropped image of the western blot is presented on Supplementary information.

To examine the effect of hypobaric hypoxia on the levels of Nrf2/HO-1, we explored Nrf2 mRNA through real-time PCR analysis. We observed that the relative Nrf2 mRNA expression was dramatically decreased in the hypobaric hypoxia group. Conversely, HSD intervention elevated hypobaric hypoxia stress-initiated down-regulation of Nrf2 when compared to the hypoxia group, indicating that HSD ameliorates oxidative stress-caused retinal impairment (Fig. 1A).

Quantification of HO-1 mRNA revealed that hypobaric hypoxia significantly inhibited mRNA expression of HO-1 when compared to the unstimulated control retina. On the contrary, the HSD treatment reverted this effect by substantially upregulaing HO-1 mRNA expression in the retina (Fig. 1B).

The western blot analysis showed that hypobaric hypoxic exposure resulted in a notably decrease in Nrf2 and HO-1 protein levels in the retina compared with the control (Fig. 1C–E). HSD intervention mitigated the hypobaric hypoxia-triggered down-regulation of Nrf2 and HO-1 protein levels (Fig. 1C–E). Our result implies that HSD administration protects retinal function from oxidative stress through enhancement of Nrf2/HO-1 expression.

### HSD inhibited hypobaric hypoxia-induced retinal cell apoptosis

**Figure 2:**
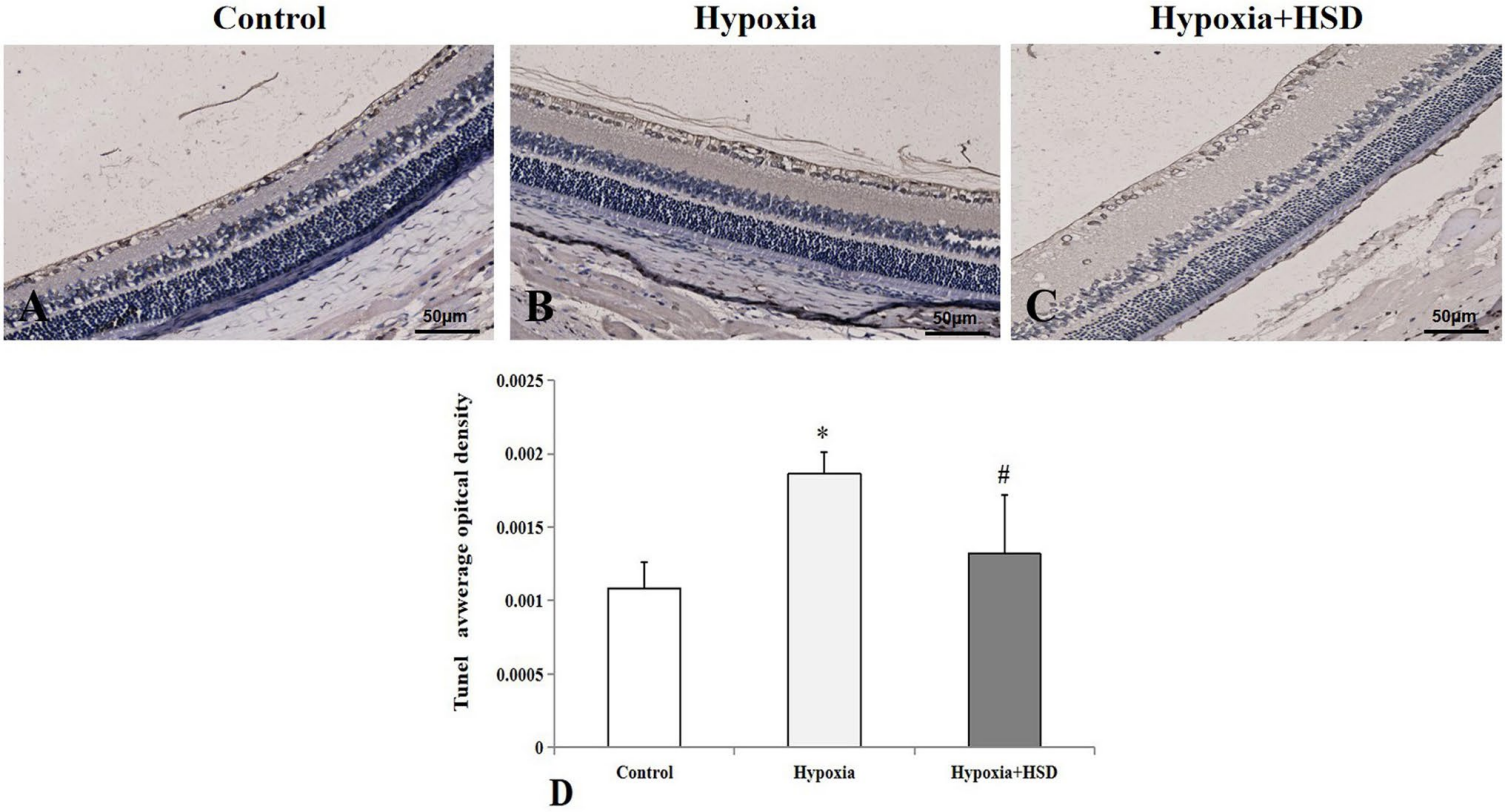
Representative micrographs of TUNEL-stained retinal sections. (**A**) Control group; (**B**) Hypobaric hypoxia group; (**C**) Hesperidin intervention (Hypoxia + HSD) group; (**D**) Quantitative analysis of TUNEL-positive average opitcal density in the retina (mean ± SEM, n = 4). *p < 0.05, versus control; ^#^*p* < 0.05, versus hypobaric hypoxia group. Scale bar = 50 μm.

With a view to exploring the underlying mechanisms being responsible for the improvement of retinal functions following HSD administration in hypobaric hypoxia condition, we performed TdT-mediated dUTP nick end labeling (TUNEL) staining for the detection of DNA fragmentation. After hypobaric hypoxia exposure, TUNEL-positive cells in retinal ganglion cell (RGC) layer increased as compared with the normoxia group (Fig. 2A,B,D). HSD significantly decreased TUNEL positive expression (Fig. 2C,D), suggesting that intervention with HSD protects retinal cells against hypobaric hypoxia-induced damage.

### HSD administration reduced hypobaric hypoxia-induced apoptotic caspase up-regulation, preserved antiapoptotic protein Bcl-2 and suppressed the proapoptotic protein Bax level

**Figure 3:**
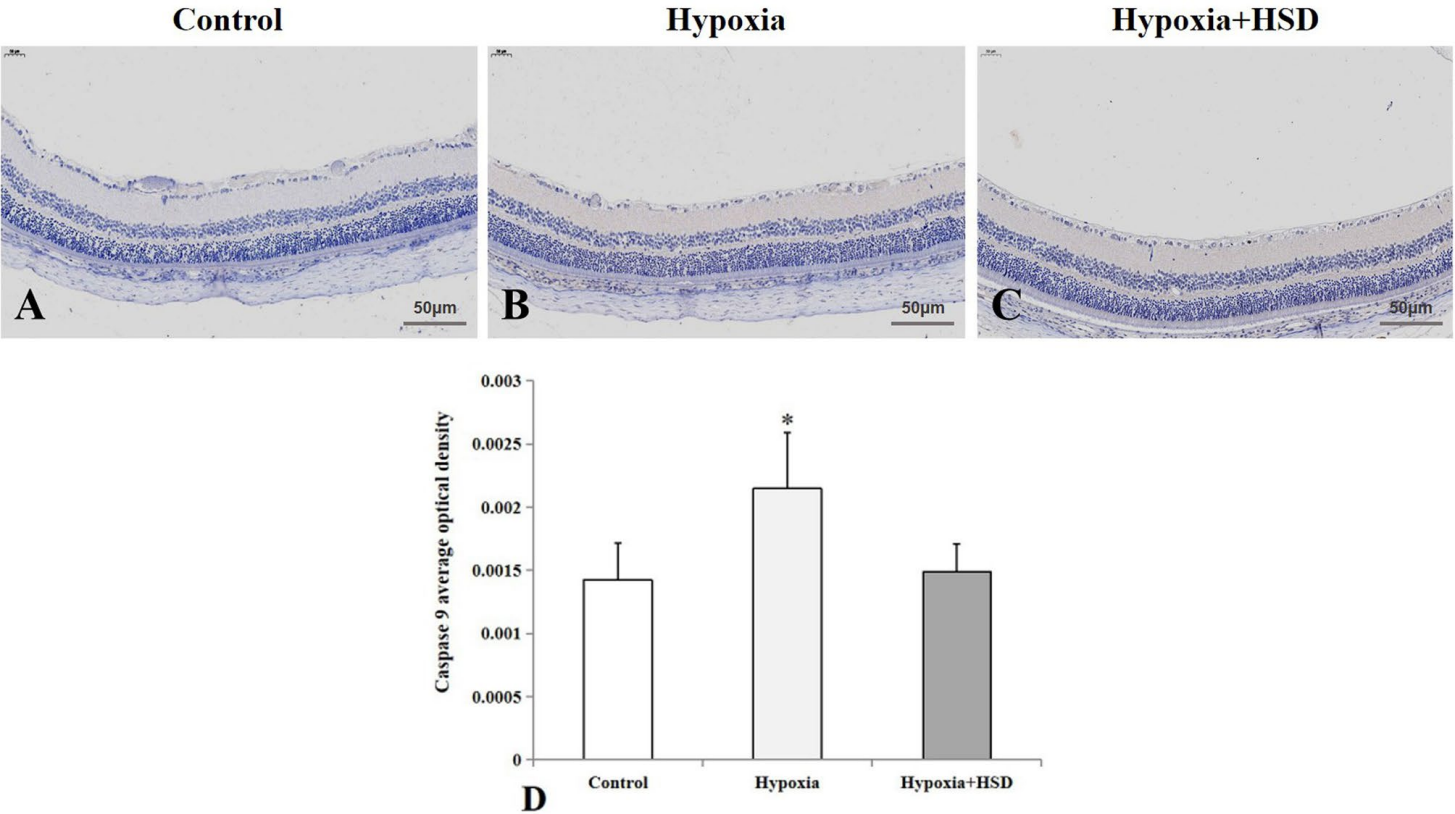
Immunostaining of caspase9 expression in rat retina. (**A**) Control group; (**B**) Hypobaric hypoxia group; (**C**) Hesperidin intervention (Hypoxia + HSD) group; (**D**) Positive immunostaining intensity of caspase9 expression. Results are presented as mean ± SEM (n = 4 per group). *p < 0.05, versus control. Scale bar = 50 μm.

**Figure 4:**
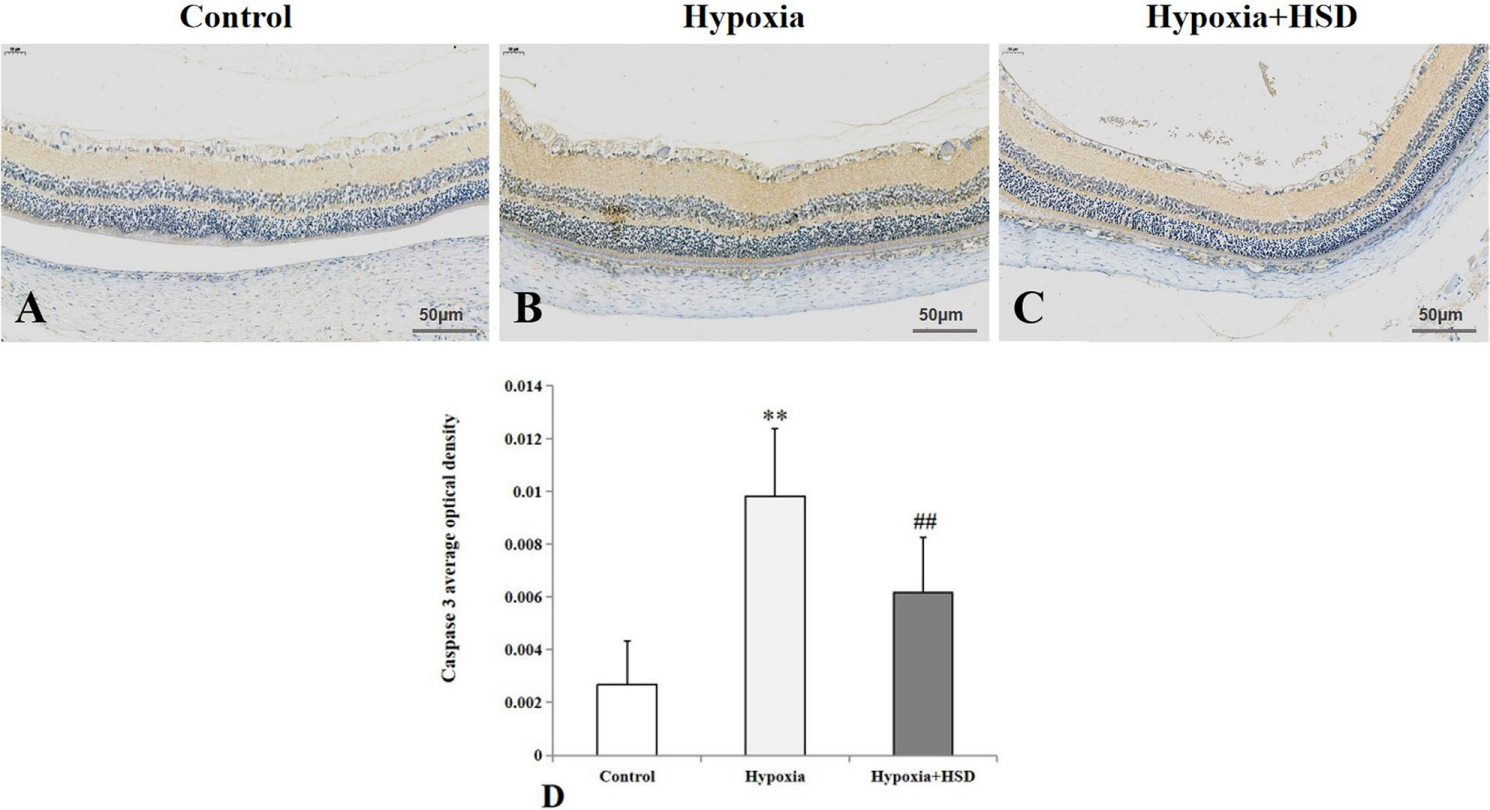
Immunostaining of caspase3 expression in rat retina. (**A**) Control group; (**B**) Hypobaric hypoxia group; (**C**) Hesperidin intervention (Hypoxia + HSD) group; (**D**) Positive immunostaining intensity of caspase3 expression. Results are presented as mean ± SEM (n = 4 per group). **p < 0.01, versus control; ^##^*p* < 0.01, versus hypobaric hypoxia group. Scale bar = 50 μm.

Exposure to acute hypobaric hypoxia condition increased caspase9 positive immunostaining in the retinal inner plexiform layer and outer plexiform layer (Fig. 3A,B,D). HSD intervention suppressed caspase9 expression in the retina as compared to the hypoxic samples; however, this result did not reach statistical significance (Fig. 3B–D). As shown in Fig. 4, strong caspase3 positive immunoreactivity was presented in the retinal nerve fiber layer, retinal ganglion cell layer, inner plexiform layer and outer plexiform layer as well as photoreceptor layer under hypoxic condition. HSD treatment notably ameliorated caspase3 expression level in the retina compared with the hypoxia group.

**Figure 5:**
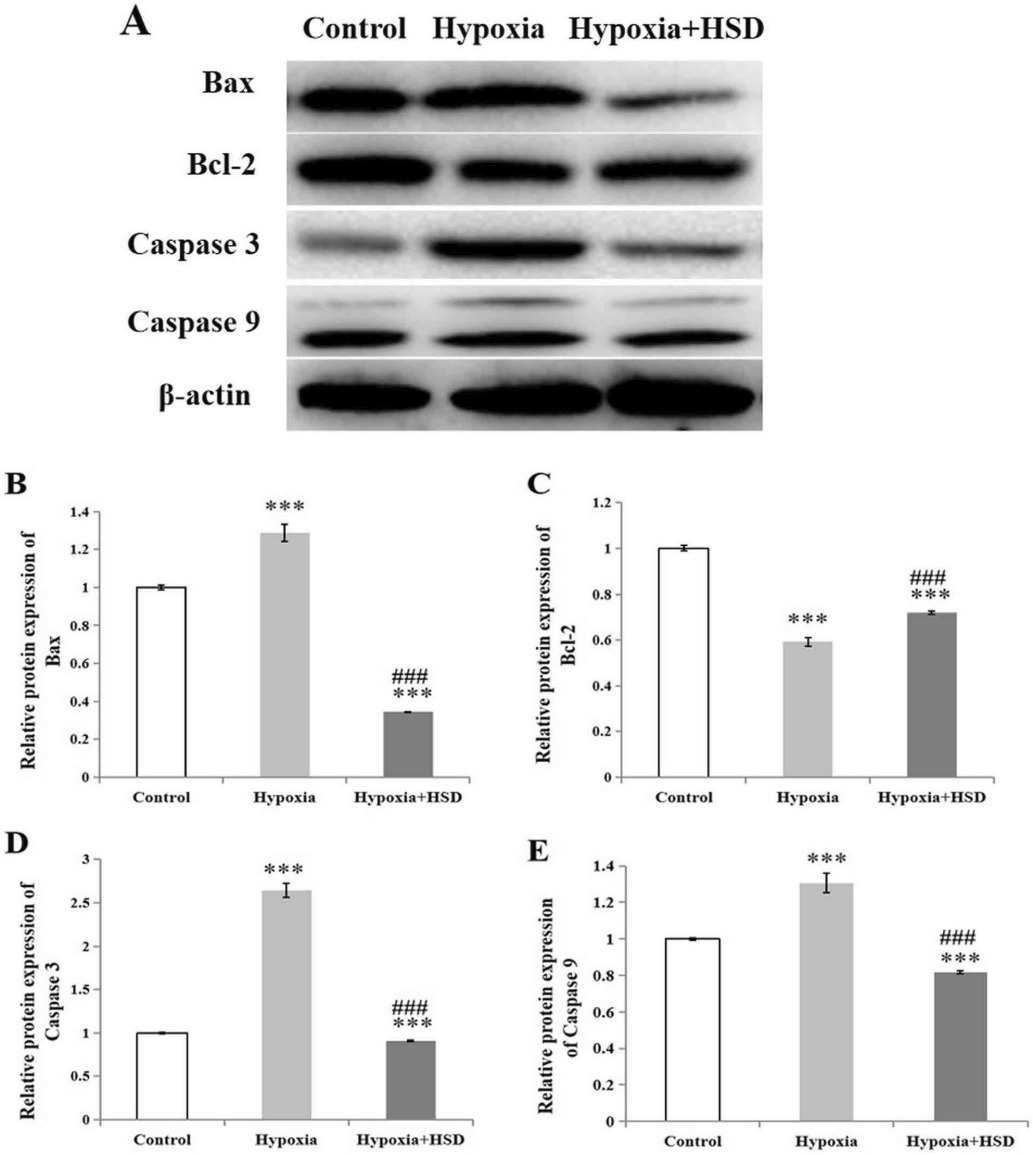
Western blot analysis for proteins. (**A**) The protein levels of Bax, Bcl-2, caspase9 and caspase3 were evaluated by western blot analysis. β-actin was used to ensure equal loading. (**B**–**E**), Densitometric analysis of proteins (B:Bax; C:Bcl-2; D:caspase3; E:caspase9). Data were shown as mean ± SEM (n = 6 ),***p < 0.001, versus control; ^###^*p* < 0.001, versus hypobaric hypoxia group. The uncropped image of the western blot is presented on Supplementary information.

Considering the significant changes observed in the retina of the hypoxia stimulation, we also analyzed the expression levels of apoptosis-related proteins by western blot. We found that Bax, the mitochondrial related pro-apoptotic protein, increased under oxidative stress in comparison with that in the control group. HSD intervention significantly reduced Bax protein level (Fig. 5A,B). Mitochondrial related anti-apoptotic protein of Bcl-2 in the normoxia and HSD treatment groups were elevated compared with the hypobaric hypoxia group (Fig. 5A,C). The western blot results showed that hypobaric hypoxic environment exposure resulted in a markedly increase in caspase9 and caspase3 expression in retina compared with the control (Fig. 5A,D,E). HSD intervention reduced hypobaric hypoxia-induced caspase9 and caspase3 protein level elevation (Fig. 5A,D,E). These results strongly indicate that HSD treatment exerts an anti-apoptosis function by suppressing apoptotic caspases expression, reducing Bax level and preserving Bcl-2 expression in response to the hypobaric hypoxia stress.

### HSD declined poly(ADP-ribose) polymerase 1 (PARP1) production in the retina against hypobaric hypoxia stress

**Figure 6:**
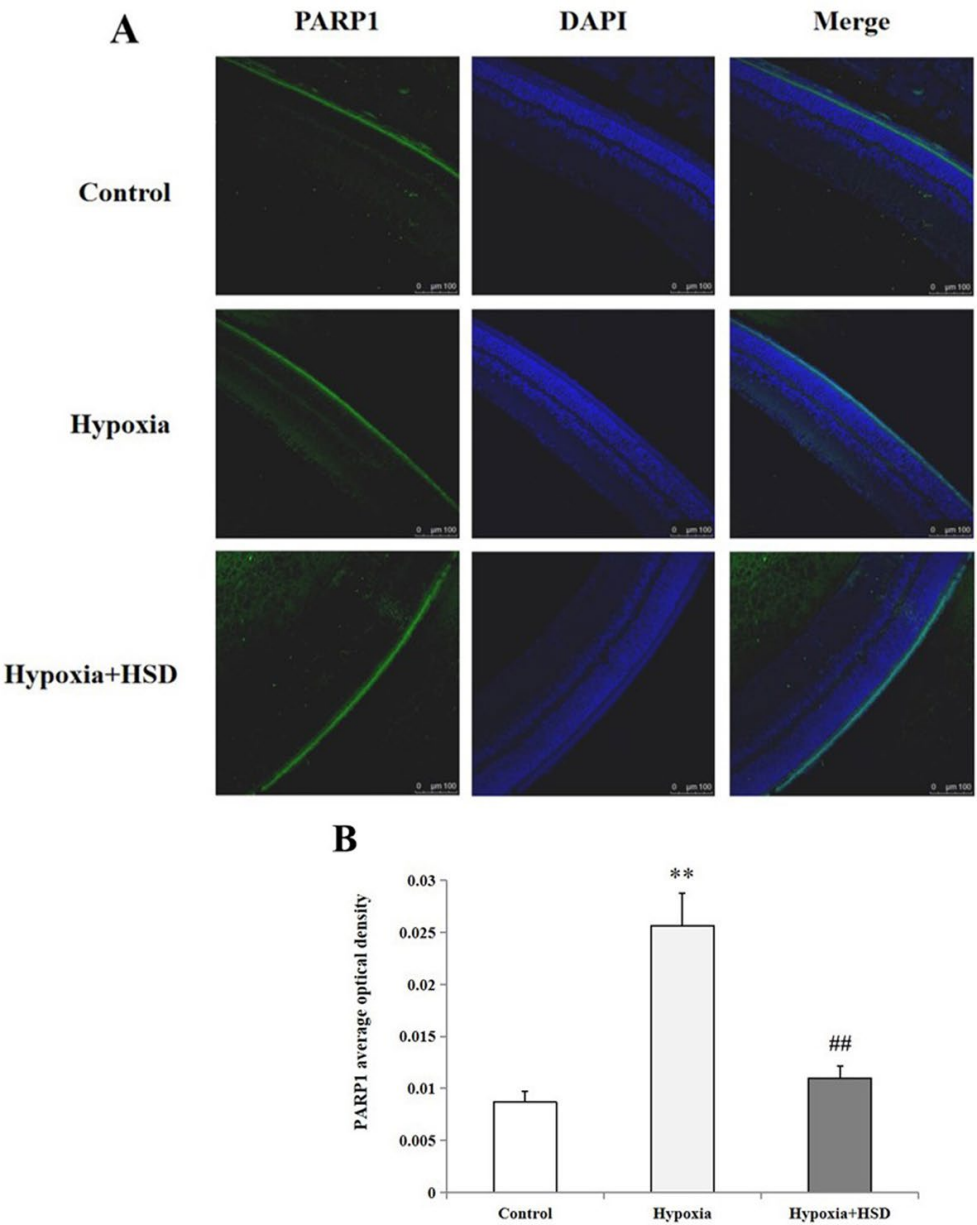
(**A**) Micrographs of PARP1 immunofluorescence staining in rat retina of control, hypobaric hypoxia and hesperidin intervention (HSD + hypoxia) group; (**B**) Positive immunostaining intensity of PARP1 expression. Results are presented as mean ± SEM (n = 4 ). **p < 0.01, versus control; ^##^*p* < 0.01, versus hypobaric hypoxia group. Scale bar = 100 μm.

We determined whether hypobaric hypoxia stress could stimulate the expression of PARP1 in the rat retina. We observed that increased positive PARP1 influorescence exhibited in the outer plexiform layer and inner plexiform layer in the hypoxia group compared with the normoxia group. The influorescence intensity was ameliorated when HSD was applied in hypobaric hypoxic condition (Fig. 6).

### HSD mitigated hypobaric hypoxia-induced ciliary neurotrophic factor (CNTF) down-regulation

**Figure 7:**
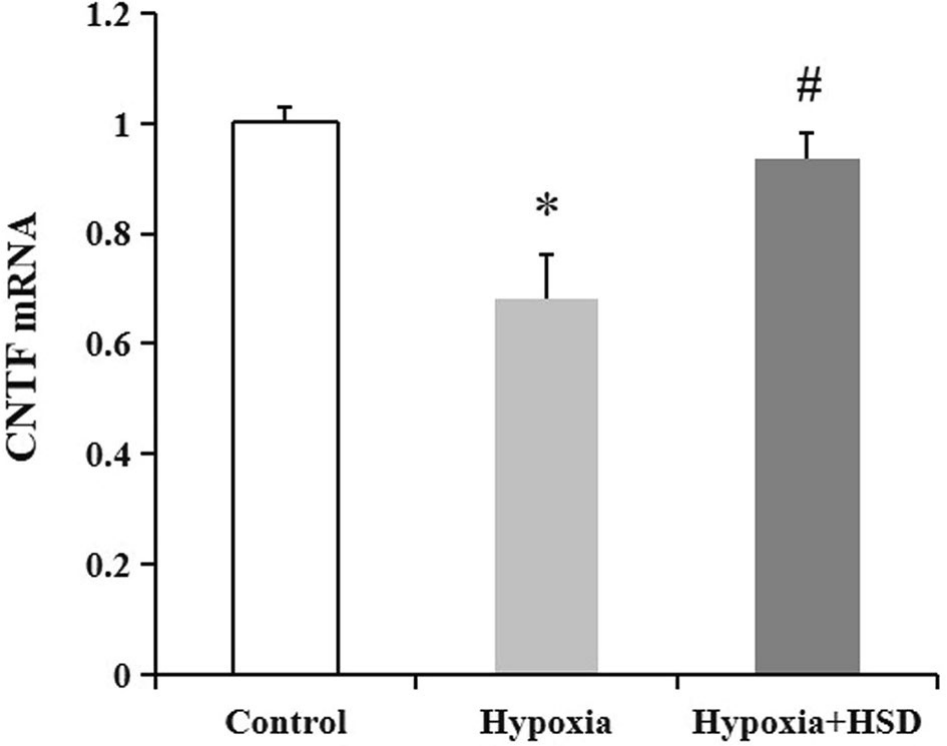
CNTF mRNA expression in rat retina of control, hypoxia and hesperidin intervention (Hypoxia + HSD) groups. Results are presented as mean ± SEM (n = 6). *p < 0.05, versus control; ^#^*p* < 0.05, versus hypobaric hypoxia group.

We analyzed the mRNA expression of CNTF, a neuron survival promote motor^13,14^, response to hypobaric hypoxia, and also evaluated the contribution of HSD to CNTF mediation. CNTF expression analysis through RT-PCR revealed that CNTF mRNA downregulated in the hypobaric hypoxia-exposure group relative to the control, whereas HSD administration significantly alleviated the oxidative impairment through elevating CNTF expression in comparison to the hypobaric hypoxia group (Fig. 7).

## Discussion

In the present study, we used the model of hypobaric hypoxia that mimics the condition of 5000 m altitude exposure. We observed that hypobaric hypoxia induced a significant decrease in Nrf2 expression. A similar response was found in HO-1 level after exposure to the oxidative stress. Nrf2 has been demonstrated to be exploited as a regulator transcription factor involved in modulating cellular redox balance and promoting the expression of antioxidant enzymes against oxidative damage^15^. Nrf2 is sequestered in the cytoplasm by binding to the cysteine- (Cis-) rich Kelch-like ECH-associated protein 1 (Keap1) under normal condition and plays a key role in detoxification of reactive oxidants, maintenance of cellular homeostasis, removal of numerous exogenous and some endogenous chemicals^15, 16^. Under the oxidative irritation, phosphorylated Nrf2 activates key downstream targets including HO-1 along with other antioxidant proteins after its release from Keap1 and translocation into the nucleus through binding to the antioxidant responsive elements^17–19^. HO-1 is one of the cytoprotective genes and exerts critical functions in mediating antioxidant, anti-inflammatory, and anti-apoptotic effects^20–22^. Furthermore, enhancement of HO-1 expression after ischemic stress may extend neuronal survival^23^. Our study demonstrated that HSD administration attenuated hypobaric hypoxia-induced oxidative damage through elevation of Nrf2/HO-1 expression. The results presented here imply that HSD is capable of activating the Nrf2/HO-1 pathway to ameliorate hypobaric hypoxia-caused oxidative stress.

Our findings demonstrated that hypobaric hypoxia resulted in increased programmed cell death in the rat retina analyzed by TUNEL assay. We further explored the effect of HSD on hypobaric hypoxia-induced apoptosis. We found that HSD intervention plays an important role in mitigating apoptosis initiated by hypobaric hypoxia. The apoptotic process can be triggered by the intrinsic and the extrinsic pathways. Caspase9 and caspase3 function as the key enzymes involving in the mitochondria-dependent intrinsic apoptosis pathway^24–26^. Caspase9 belongs to initiator caspases to induce apoptosis via activating the downstream caspase3, which is one member of effector caspases. Our results revealed a significant increase in protein levels of caspase9 and caspase3 upon hypobaric hypoxia stress. These results are consistent with our previous study findings in which hypoxic condition elevated caspase9 and caspase3 mRNA expression in the rat retina, further suggesting that hypobaric hypoxia induces apopotosis through the intrinsic mitochondrial pathway^7^. HSD supplementation inhibited the hypobaric hypoxic stress-induced intrinsic apoptosis by decreasing protein levels of both caspase9 and caspase3.

Additionally, our results revealed that a reduction in the Bcl-2 expression and an increase in Bax level following hypobaric hypoxia exposure. HSD treatment reversed this result by remarkably enhancing Bcl-2 expression and decreasing Bax level. The Bcl-2 family proteins are considered as crucial regulators in the intrinsic or “mitochondrial” apoptosis pathway. As a member of the Bcl-2 family, Bcl-2 plays a pivotal role in the cellular defense against oxidative stress and apoptosis. The proapoptotic gene of Bax is vital in mitochondrial-mediated apoptosis in neuronal cell types^27^. Bax deficiency in mice resulted in more RGCs surviving to prevent RGCs apoptosis after optic nerve crush and axotomy^28^. Our findings suggest that HSD protects the retinal function via Bax down-regulation and Bcl-2 up-regulation against the mitochondria-related apoptotic pathway in hypoxic retinal injury. So a significant difference between hypobaric hypoxia condition and HSD intervention on caspase9, caspase3, Bax and Bcl-2 expression as well as DNA fragmentation formation in our study further supports an anti-apoptosis capacity of HSD in response to hypobaric hypoxia stress in the rat retina. It has been shown that Nrf2/HO-1 cascade inhibits apoptosis based on its effect on mitochondria-related apoptotic proteins such as Bcl-2 and Bax^29^. The protective role of HSD in mitigating hypobaric hypoxia-induced apoptosis in the rat retina may be in part related to its activation of Nrf2/HO-1 pathway through up-regulation of Nrf2 and HO-1 expression.

PARP1 functions as a nuclear enzyme which plays an essential role in regulating DNA repair, cellular metabolism, transcription, chromatin modulation, DNA replication, and apoptosis^30,31^. PARP1 is therefore crucial for the survival and homeostasis of cells. Emerging evidence has linked the regulatory effects of PARP1 to the modulation of inflammation, metabolism, circadian rhythms, and cancer^32^. A key finding in our study showed that hypobaric hypoxia triggered the activation of PARP1. PARP1 is considered as a DNA damage sensor by binding to impaired DNA, consuming NAD^+^ and inducing ATP decrease, and consequently leading to cellular energetic depletion, mitochondrial dysfunction and necrosis^33,34^. Hypobaric hypoxia stress-induced apoptosis in our study may be partially attribute to the participation of PARP1 activation, which causes mitochondrial damage and initiates the “mitochondrial” apoptosis pathway^35^. We found that HSD is capable of regulating PARP1 activity by reversing the hypobaric hypoxia-initiated PARP1 up-regulation. This result suggests that HSD may deactivate PARP1 to preserve cellular NAD levels and thus retain its own function. Our data imply a protective role of HSD in stress condition through blocking the PARP1 over-activity in the hypobaric hypoxia stress.

It is well accepted that CNTF functions as a neuroprotective agent by promoting motor neuron survival, inhibiting microglial activation and attenuating microglia-derived oxidative stress^13,36^. Various glial cells especially astrocytes express CNTF in the neural retina and optic nerve head^37–39^. Several in vitro studies have shown that CNTF is capable of protecting both photoreceptors and ganglion cells from apoptosis^40–43^. The loss of neurotrophic properties of CNTF is correlated with retinal diseases in several animal models^43–45^. It has been reported that there was a reduction of CNTF concentration in the blood serum in patients with primary open–angle glaucoma; therefore, CNTF has a potential in stimulating axon regeneration and promoting axonal survival^46^. Our data demonstrated that hypobaric hypoxia reduced endogenous CNTF level. This decreased endogenous neurotrophic response may be not sufficient to protect the injured retina, whereas HSD administration significantly ameliorated the oxidative stress through enhancing CNTF expression. CNTF has been shown to activate Nrf2 signaling and inhibit oxidative stress^47^. The present findings including the elevation of CNTF and Nrf2 as well as HO-1 in HSD intervention condition suggest that HSD improves CNTF release to exert and enhance its neuroprotective effects probably by activating Nrf2/HO-1 pathway against the oxidative interruption.

The findings of the current study indicate that HSD is instrumental in minimizing hypobaric hypoxia-associated retinal impairment by activating the Nrf2/HO-1 antioxidant pathway, anti-apoptosis, suppressing PARP1 and upregulating CTNF expression in the rat retina. Based on these observations, HSD may have a great potential to be a promising intervention for attenuating the hypobaric hypoxia-mediated retinal dysfunction.

## Materials and methods

Male Sprague–Dawley rats (SPF-class; weight: 200–250 g) were purchased from Experimental Animal Center of Xian Communication University (certificate number: SCXK Shanxi 2018–001). Animals were housed in a clean environment with stable temperature and relative humidity of 55% in a 12 h light–dark cycle. Rats were adaptive maintained in the animal room for 2 weeks before the experiment. Animal procedures were approved by the ethical committee of University of Electronic Science and Technology of China and conducted in compliance with the Guide for the Care and Use of Laboratory Animals of the National Institutes of Health.

### Experimental design

SD rats were divided into three groups including control group, hypoxia group, and HSD intervention group. The hypoxia group and HSD intervention group were housed in a low-pressure oxygen cabin which mimics the environment with 5000 m altitude. The HSD intervention group was applied daily intragastrical administration with HSD (40 mg/kg, Adamas-beta, China), and the hypoxia group was given the same dose of normal saline intragastrically.

### Quantitative real-time PCR

The eyes were enucleated following different treatments for 7 days, and the retinas were collected and kept at − 80 °C. RNAiso Plus reagent (TaKaRa, Shiga, Japan) was used to extract the total RNA. Reverse transcription was performed with PrimeScript RT Master Mix (TaKaRa, Shiga, Japan). Real-time PCR was worked with Power SYBR Green PCR Master Mix (Thermo Fisher Scientific Inc., USA). All reactions were conducted on StepOnePlus for 40 cycles. Relative gene expression was normalized by the median expression of GAPDH as an internal control. The primers utilized for real-time PCR were listed in Table 1.

**Table 1:** List of primers used for quantitative real-time PCR.

Primer	Forward (5′—3′)	Reverse (5′—3′)
Nrf2	TTTGTAGATGACCATGAGTCGC	GCCAAACTTGCTCCATGTCC
HO-1	ACAGAAGAGGCTAAGACCGC	GAGCGGTGTCTGGGATGAAC
CNTF	ATGGCTTTCGCAGAGCAAAC	CGGTAAGCCTGGAGGTTCTC
GAPDH	AGACAGCCGCATCTTCTTGT	CTTGCCGTGGGTAGAGTCAT

### TUNEL assay

Eyeballs were fixed in 4% paraformaldehyde solution after rats were euthanized following different treatments for 7 days. Apoptotic activity within the retina was determined using TUNEL assay kit (Abcam, USA). The retinal cross sections were deparaffinized, rehydrated and washed with PBS. Endogenous peroxidase activity was blocked by 0.3% hydrogen peroxide solution (H_2_O_2_) for 10 min. The sections were washed in PBS and TdT reaction enzyme was added and quenched with stop/wash buffer. Anti-digoxigenin-peroxidase conjugate was added and incubated in a humidified chamber. The reaction was observed with 0.06% 3,3′-diaminobenzidine tetrahydrochloride (Sigma-Aldrich, USA) staining. Sections were counterstained, mounted, and visualized under a microscope (Olympus IX73, Japan).

### Western blot

The retinas were ultrasonically homogenized at 4 °C in RIPA buffer (Beyotime, China). Protein was separated by SDS-PAGE and transferred to polyvinylidene difluoride (PVDF) membranes (Millipore Corporation, USA). The membranes were blocked with 5% non-fat milk and 0.1% Tween-20 in PBS for 1 h, followed by incubation with anti-Bcl-2 antibody (1:1000; Proteintech Group, USA), anti-Bax antibody (1:5000; Proteintech, USA), anti-caspase3 antibody (1:1000; Proteintech, USA), anti-caspase9 antibody (1:1000; Proteintech Group, USA), anti-Nrf2 antibody (1:1000; Proteintech Group, USA), and anti-HO-1 antibody (1:1000; Proteintech Group, USA) overnight at 4 °C. After several washing, the membranes were incubated with horseradish peroxidase (HRP)-conjugated anti–rabbit secondary antibody (1:10,000; Jackson ImmunoResearch Inc., USA) for 1 h at room temperature. The protein bands were detected using an enhanced chemiluminescence (Millipore Corporation, USA) reagent. Image J software was used to determine the protein band intensities and quantification.

### Immunohistochemistry

Eyeball sections were deparaffinized and permeabilized using 0.05% Triton X-100. The samples was blocked by 3% bovine serum albumin and incubated with primary antibodies against caspase 3 (1:50), caspase 9 (1:100) (Proteintech Group, USA) overnight at 4 °C and washed with PBST. Sections were further incubated with anti-rabbit secondary antibody (1:500) (Jackson ImmunoResearch Inc., USA) followed by peroxidase staining with the peroxidase substrate 3,3-diaminobenzidine tetrahydrochloride. Sections were counterstained, mounted, and observed under a microscope.

For immunofluorescence staining of retinal cross sections, tissues were treated in 1% goat serum albumin/PBS for 1 h at room temperature before incubation with the primary antibody against PARP1 (1:200; Proteintech Group, USA) overnight at 4 °C. After several washes, sections were incubated with the secondary antibody Alexa Fluor 488 donkey anti-rabbit IgG antibody (1:100, Invitrogen, USA) at room temperature for 1 h and washed with PBS. The sections were counterstained with DAPI (Invitrogen, USA) in PBS. The immunostaining results were analyzed by Image-Pro plus 6.0 software (Media Cybernetics, Inc., Rockville, MD, USA).

### Statistical analysis

The data were expressed as mean ± SEM. Analysis between multiple groups was compared using one-way ANOVA analysis followed by Bonferroni multiple comparison post-tests. Differences were considered significant at *p* < 0.05.

## Supplementary information

Supplementary Information
