# Supplementary material for: Hesperidin ameliorates hypobaric hypoxia-induced retinal impairment through activation of Nrf2/HO-1 pathway and inhibition of apoptosis
Source: Sci Rep. 2020 Nov 10;10:19426. doi: 10.1038/s41598-020-76156-5 (PMC7655840; doi:10.1038/s41598-020-76156-5)
Supplement: Supplementary file 1 — Supplementary Information [file 41598_2020_76156_MOESM1_ESM.pdf]

Supplementary information

Hesperidin ameliorates hypobaric hypoxia-induced retina impairment through activation of Nrf2/HO-1 pathway and inhibition of apoptosis

Xiaorong Xin, Yanrong Li, Haiping Liu

Department of Ophthalmology, Sichuan Provincial People's Hospital, University of Electronic Science and Technology of China, Chengdu 610072, Sichuan Province, China

Author information

Affiliations

Department of Ophthalmology, Sichuan Provincial People's Hospital, University of Electronic Science and Technology of China, Chengdu 610072, Sichuan Province, China

Xiaorong Xin, Yanrong Li, Haiping Liu

Corresponding author

Correspondence to **Xiaorong Xin** xrgc19@yahoo.com

The detailed information of original western blot for Figure 1C.

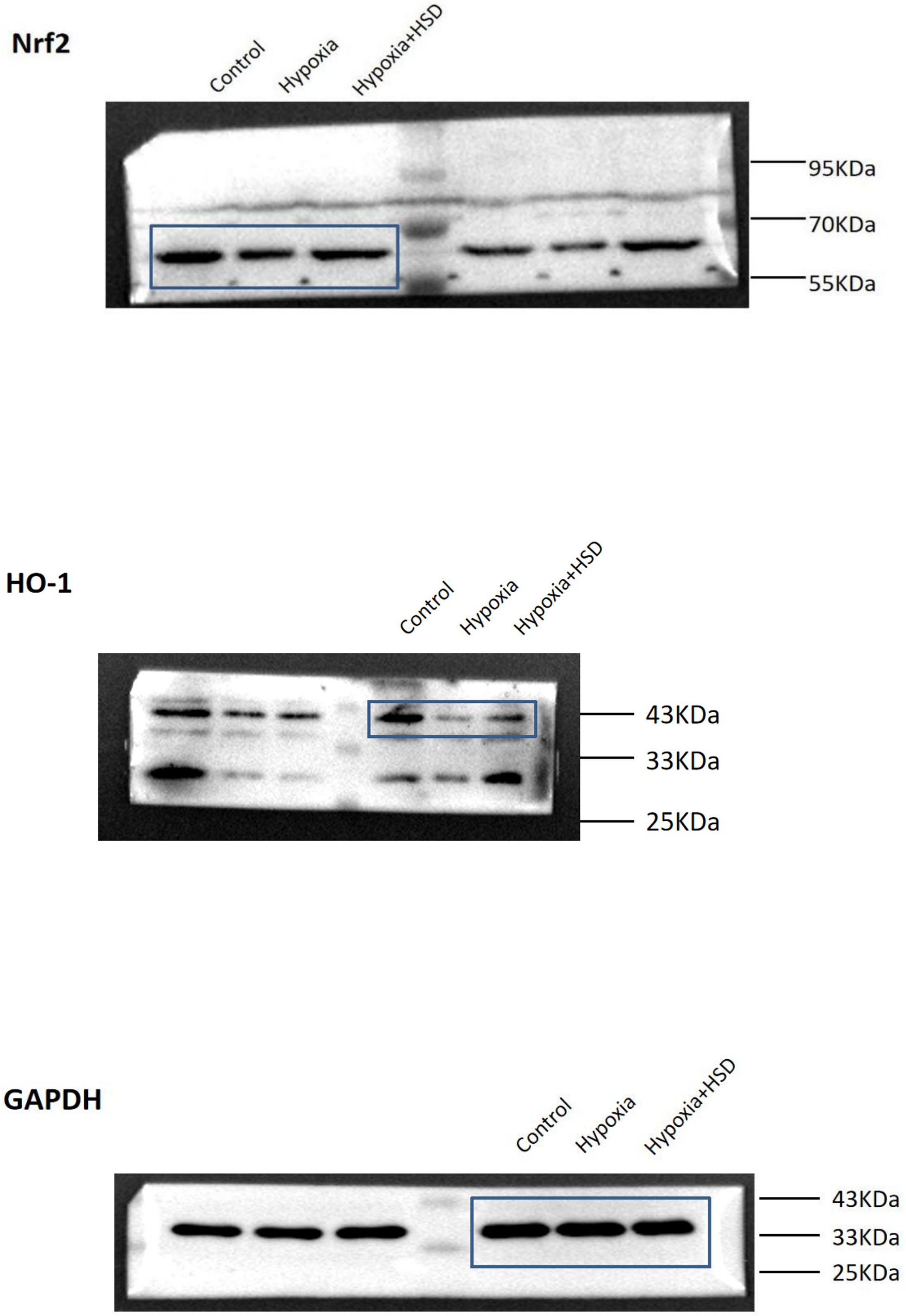

Supplementary Figure. S1. The full blot images, with molecular weight markers indicated for Figures 1C.

The detailed information of original western blot for Figure 5A.

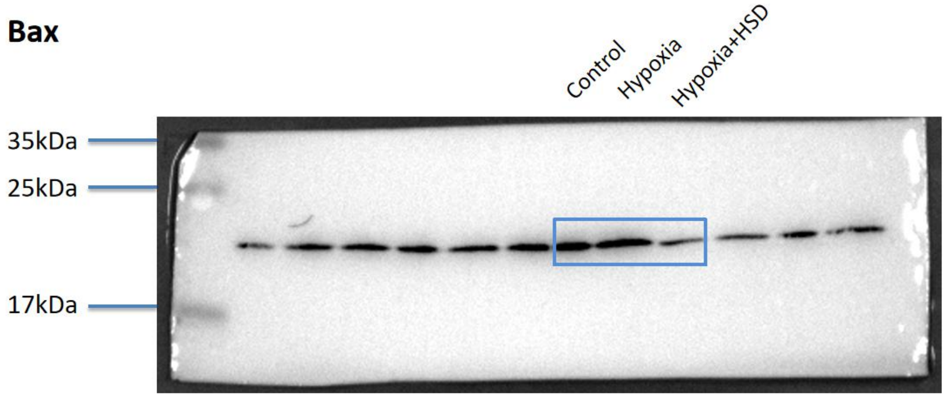

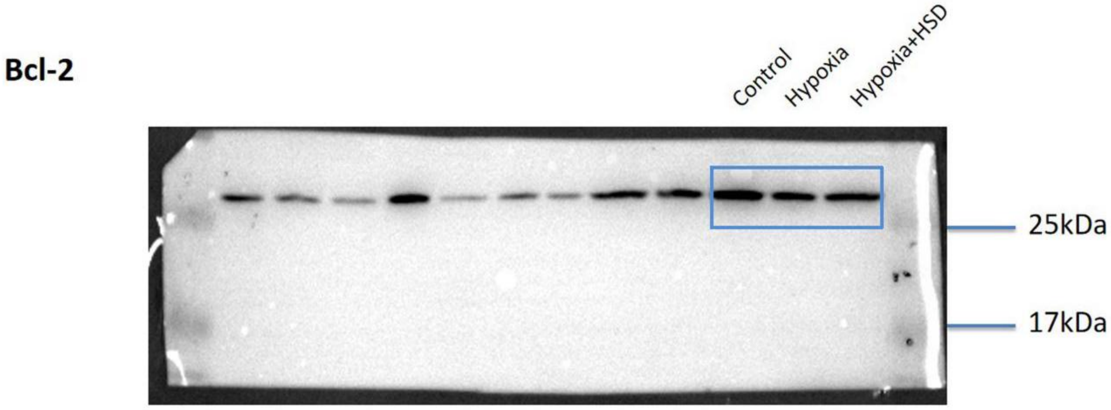

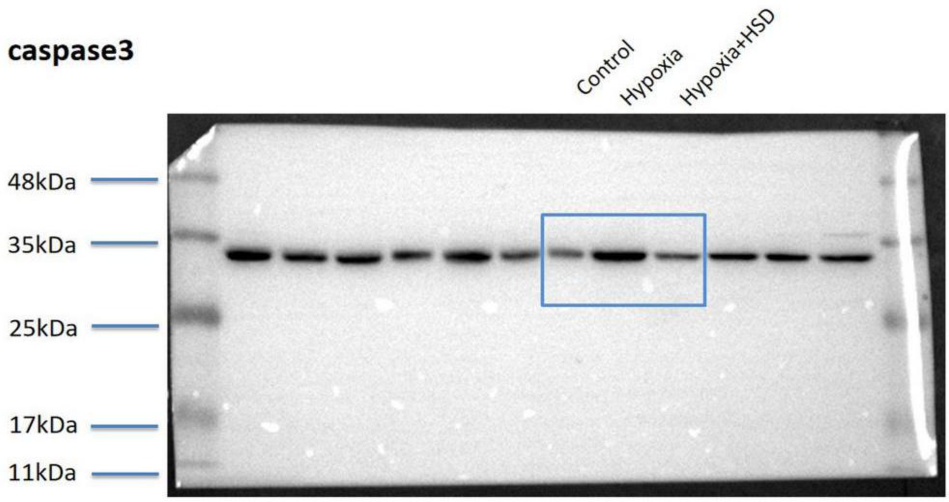

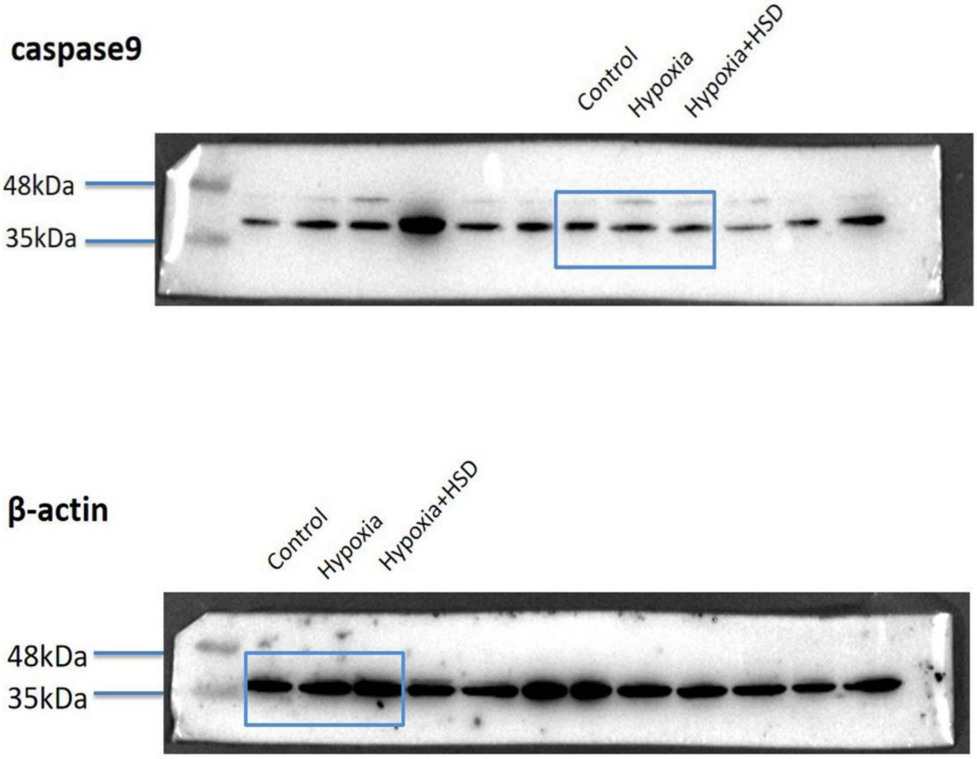

Supplementary Figure. S2. The full blot images, with molecular weight markers indicated for Figures 5A.
